# L-asparaginase production in the pseudomonas pseudoalcaligenes strain JHS-71 isolated from Jooshan Hot-spring 

**Published:** 2016-03

**Authors:** Arastoo Badoei-Dalfard

**Affiliations:** Department of Biology, Faculty of Sciences, Shahid Bahonar University of Kerman, Kerman, Iran

**Keywords:** L-Asparaginase, *Pseudomonas*, Sirch hot-spring, Screening, Identification

## Abstract

L-asparaginase has lots of medical and industrial applications. Ever since L-asparaginase anti-tumor activity was first demonstrated, its production using microbial systems has attracted considerable attention owing to their cost-effective and eco-friendly nature. The aim of this study is to obtain L-asparaginase producing bacteria and determining the enzyme activity. Samples were picked up from Jooshan hot springs located in the Sirch, Kerman. The L-asparaginase producing bacteria were screened on the agar medium supplied with L-asparagine and phenol red indicator dye (pH-7.0). L-asparaginase activity was detected on the basis of pink color around the colony. Enzyme production was also performed based on ammonia detection by Nessler method. Among 24 strains, there were 7 strains which could produce L-asparaginase. Sequencing of 16S rRNA showed that, the best isolates producing L-asparaginase belongs to the* Pseudomonas *genus. Enzyme activity after 24 and 48 h of incubation showed that *Pseudomonas pseudoalcaligenes *strain JHS-71 was the best strain that produced L-Asparaginase about 240 (U/ml) after 48h of incubation. Results showed that, L-Asparaginase activity enhanced about 27% in the presence of Co^+2^. L-asparaginase JHS-71 retained more than 50% of its initial activity in the presence of Cu^+2^, Mn^+2^, Zn^+2^, Mg^+2^ and Fe^+2^. Because of various applications of L-asparaginase in biotechnology, *P. pseudoalcaligenes* strain JHS-71 can be used as a suitable candidate in these fields.

## INTRODUCTION

**Figure1:**
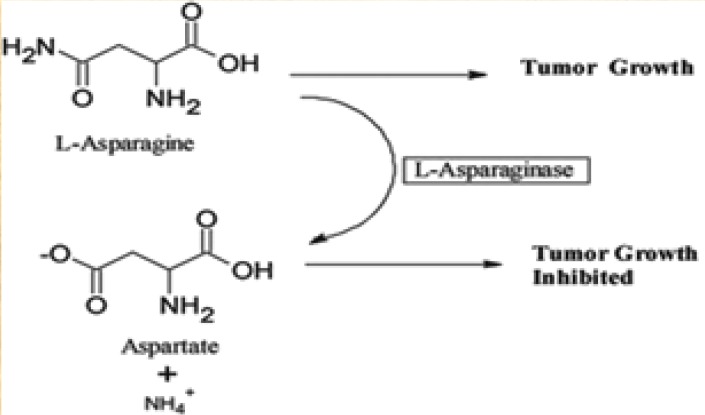
Schematic illustration of the reaction mechanism of L-asparaginase

L-asparaginase catalyzes the hydrolysis of L-asparagine amino acid into L-aspartic acid and ammonia [1]. In general, L-Asparaginase has two applications, one of them is in the nutrition production [2], where L-asparaginase is used to make french fries and potato chips [3]. This is performed to diminish the acrylamide formation which takes place at high temperatures of frying or baking as a result of Millard reaction between reducing sugars and L-Asparagine [3, 4]. The anticancer property of this enzyme in the treatment of acute lymphoblastic leukemia is the other application of this enzyme [1]. Cancer cells need L-asparagine while they cannot make enough L-asparagine owing to very low levels of L-asparagine synthetase. Therefore, they are dependent on the serum levels of L-asparagine for their survival and propagation [5-8]. Administration of L-asparaginase withdraws dependent of cancer cells to the extracellular source of L-asparagine (Fig. 1). Conversely, healthy cells escape natural as they are capable of producing L-asparagine with L-asparagine synthetase [9-11].

The L-asparaginase activity was first reported by Lang (1904) and further established by Furth & Friedmann (1910) and Clementi (1922) [1, 12]. The prospective of the L-asparaginase in cancer was first discovered by Kidd (1953), who detected the anti-lymphoma activity of the guinea pig serum [13]. Various bacteria, such as, *Bacillus subtilis* 168 [14], *Erwinia carotovora* [15, 16], *Escherichia coli* [17)-], *Thermus thermophiles* [18], *Pseudomonas aeruginosa* [19], *Enterobacter aerogenes* [20], *Zymomonas mobilis* [21], *Corynebacterium glutamicum *[22], *Erwinia Chrysanthemi *[23-25] have been found to produce L-asparaginase. Up to now, only L-asparaginase from *Erwinia* and *E. coli* has been used in the medical application. It has been reported that, *Erwinia* L-asparaginase showed less allergic responses compared to the *E. coli* L-asparaginase. Conversely, *Erwinia* asparaginase had a shorter half-life than *E. coli* asparaginase [26], offering the requisite to find new L-asparaginases that are serologically varied but have similar therapeutic properties. This may implicate the sample screening from diverse places for isolation of potential bacteria, which have the capacity to produce the anticipated L-asparaginase.

## MATERIALS AND METHODS

All materials were provided from Merck (White house Station, New Jersey, United States). The culture media ingredients were from Sigma (St. Louis, MO, USA). All other chemicals were of analytical grade and purchased from various commercial sources. The L-asparaginase producing bacteria were isolated using the modified M9 medium (1 l: 3.0 g KH_2_PO_4_; 6.0 g Na_2_HPO_4_·2H_2_O; 0.5 g NaCl; 5.0 g L-asparagine; 0.5 g MgSO_4_.7H_2_O; 0.014 g CaCl_2_.2H_2_O; 2.0 % (w/v) glucose, and 15.0 g agar) combined with a pH indicator (phenol red). Development of pink color zones around the colonies was considered as a positive result for L-asparaginase production [7]. 

Genomic DNA of *Pseudomonas *strains was extracted according to Sambrook and Russell protocols [28]. 16S rRNA PCR forward primer (5-AGT TTG ATC CTG GCT CAG-3) and reverse primer (5-GGC ACC TTG TTA CGA CTT-3) were used for the amplification of 16S rRNA genes [29]. PCR program was done as follows: (1) 94^◦^C for 5 min, (2) a run of 35 cycles with each cycle consisting of 45 s at 94^◦^C, 45 s at 48^◦^C and 90 s at 72^◦^C and (3) 10 min at 72^◦^C to permit for the extension of any incomplete products. PCR products were electrophoresed on an agarose gel (1.2%) and then DNA sequencing was accomplished on both strands directly by SEQ-LAB (Germany). The phylogenetic tree was prepared based on the comparison of 16S rRNA sequences of *Pseudomonas *strains with the other strains of *Pseudomonas* species that were obtained from the Gene Bank database (http://www.ncbi.nlm.nih.gov). All sequences were aligned with Clustal Omega and phylogenetic tree was prepared in MEGA5 [30]. 

The quantity of L-asparaginase produced was measured using 1 % L-asparagine as the substrate. L-asparaginase activity was detected by measuring the free ammonia of L-asparagine using Nessler’s reagent as described by Imada et al., (1973) [31]. For L-asparaginase production, Erlenmeyer flasks (500 ml) containing 100 ml of culture medium (M9 broth supplemented with 1% L-asparagine) was inoculated with 2 ml (2%, v/v) of an overnight culture of each isolate, separately. The flasks were incubated at 30^◦^C on a rotary shaker at 160 rpm for 72 h. Culture broth was centrifuged at 10000 rpm for 10 min to get clear supernatant. The reaction was started by adding 500 µl of supernatant into 500 µl of 40 mM L-asparagine and 500 µl of 50 mM Tris-HCl buffer (pH 7.0) and incubated at 37°C for 20 min. The reaction was stopped by adding 500 µl of 1.5 M trichloroacetic acid (TCA). The reaction mixture was allowed to centrifugation at 12000 rpm for 10 min. After centrifugation, clear supernatant was collected. 100 µl supernatant was diluted to 3.80 ml with distilled water and treated with 100 µl Nessler’s reagent and incubated at room temperature for 10 min. The absorbance of the reaction was read at 450 nm using a UV spectrophotometer. The blank was run by adding the enzyme solution after addition of TCA [32]. 

A standard curve was prepared from various dilutions of 4 mM ammonium sulfate solution. The enzyme activity was expressed in international units. One unit of L-asparaginase is the amount of enzyme which catalyze the formation of 1μmol of ammonia per min at 37^◦^C. All the experiments were carried out in triplicate. The ammonia content of each experiment was estimated by this standard curve. All data were the average values of three repeated measurements.

The effect of different metal ions on the L*-*asparaginase activity was evaluated using Fe^2+^, Fe^3+^, Hg^2+^, Ca^2+^, Mg^2+^, Zn^2+^, Cu^2+^, K^+^, Na^+^, Co^2+^, Pb^2+^ and Mn^2+^ of 2 mM strength. The influence of inhibitors and activators was also considered using 2 mM of EDTA, Triton-X 100 and SDS. The activity was measured in the presence of the mentioned compound in assay reaction. After the mentioned time, the enzyme activity in each sample was measured and expressed as a relative activity. The untreated sample was used as control [15, 33*].*

## RESULTS

Samples were picked up from Sirch hot springs located in Kerman. A total number of 24 bacterial isolates were screened for L-asparaginase production at 37°C for 72 hours. These isolates showed L-asparaginase activity on plate method assay by pink color zones. The formation of a pink zone around the bacteria was an indication of the L-asparaginase production (Table 1). The change in color (from yellow to pink) resulted from the increase in pH due to ammonia release. Further, these isolates were subjected to the secondary screening for enzyme activity. Among them, 7 isolates showed higher activity range between 20-240 IU/ml. Biochemical and morphological tests showed that, these isolates have been characterized as the *Pseudomonas* genus. All strains were *Cocobacillus*, Gram-negative, and aerobic. They were motile and showed oxidase, catalase activity and citrate consumption was positive.

**Table1:** Comparison of pink halo around each strain

Pseudomonas strains	Pink halo (mm)
JHS-13	12±3.5
JHS-14	16±1.8
JHS-15	13±2.0
JHS-17	17±2.2
JHS-21	41±3.1
JHS-24	20±0.8
JHS-71	49±2.5

**Figure 2:**
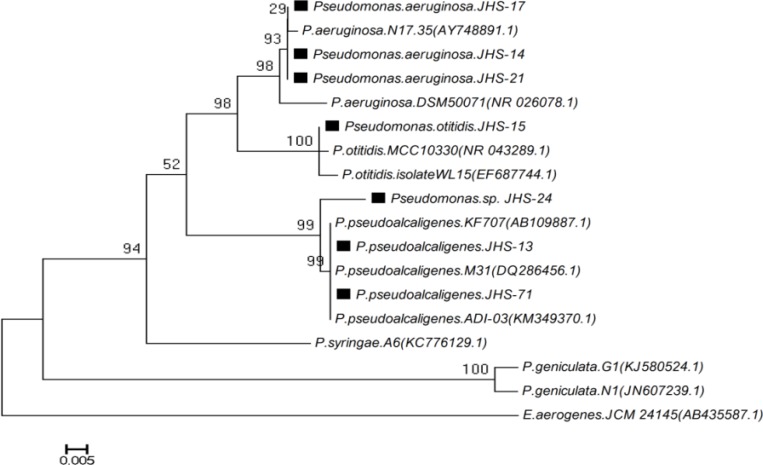
Phylogenetic tree of *Pseudomonas *strains designed by MEGA5 software. *E.aerogenes*.JCM 24145 used as out group

A total of 7 isolates were screened by plate assay on M9 media from Sirch-hot springs, located in Kerman. PCR-products showed a single band around 1500 bp on the agarose gel. 16S rRNA gene sequencing of these isolates was performed. BLAST and the phylogenetic analysis recognized that the deduced nucleotide sequences of these isolates were highly homologous with the other 16S rRNA sequences of *Pseudomonas* species. Based on the morphological, biochemical characteristics and phylogenetic analysis, these isolates were identified as follows: JHS-15 (*Pseudomonas otitidis), *JHS-13 and JHS-71* (Pseudomonas pseudoalcaligenes*)*, *JHS-14, JHS-17 and JHS-21* (Pseudomonas aeruginosa), *JHS-24 (*Pseudomonas sp.*) (Fig. 2).

**Figure 3:**
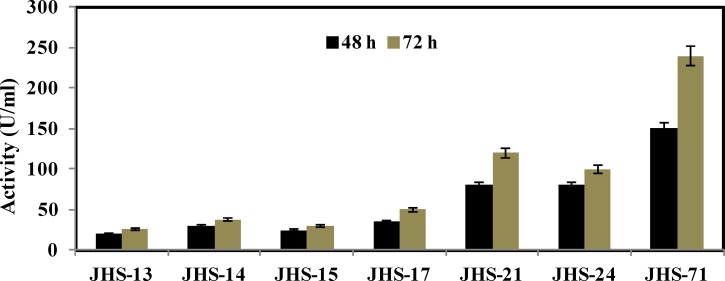
Comparison of L-asparaginase activity of *Pseudomonas *strains after 24 and 48 h of incubation in liquid media

L-asparaginase production of these isolates was examined in liquid media. Samples were picked up after 24 and 48 h of incubation and L-asparaginase activity was measured as described in the Material and Methods section. Results in Figure 3, showed that the L-asparaginase production was slightly enhanced after 48 h of incubation, compared to 24 h of incubation. These results indicated that, JHS-71 was the best L-asparaginase producer, which produced about 240 (U/ml) L-asparaginase after 48 h of incubation. In contrast, JHS-13, JHS-14, and JHS-17 produced low level of L-asparaginase, about 40 (U/ml).

**Figure 4:**
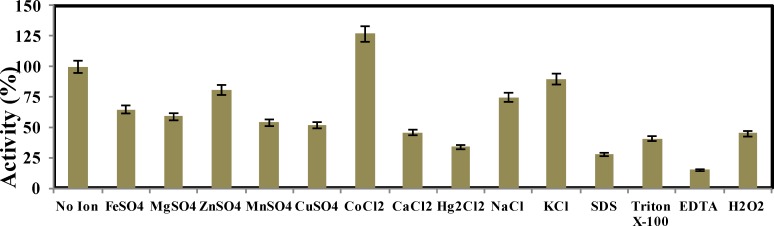
L-Asparaginase activity of *P. pseudoalcaligenes* strain JHS-71 in the presence of different compounds

In order to estimate the effects of some metal ions and compounds on the L-asparaginase activity, enzyme assay was carried out in the presence of the substrate along with each compound. The effect of several metal ions at a concentration of 2 mM on the L-asparaginase activity is shown in Figure 4. In the presence of Co^+2^, L-asparaginase activity was improved about 27%. L-Asparaginase JHS-71 retained more than 50% of its initial activity in the presence of CuSO_4_, MnSO_4_, ZnSO_4_, MgSO_4_ and FeSO_4_. The lowest enzyme activity was detected in the presence of EDTA about 15%.

## Discussion

L-asparaginase is an actual drug for the treatment of lymphoblastic leukemia and Non-Hodgkin's lymphoma. Bacteria have been proved to be a better alternative for L-asparaginase production. *Escherichia coli *and *Erwinia *strains are the most significant producers of this enzyme. *Erwinia* L-asparaginase showed less allergic responses compared to the *E. coli* L-asparaginase. Conversely, *Erwinia* asparaginase had a shorter half-life than *E. coli* asparaginase [26]. Therefore, L-asparaginase from new sources may have great financial impacts, which should not be discounted. In this study, seven *Pseudomonas* strains have been isolated from Sirch-hot spring. These strains showed good L-asparaginase activity on M9 media supplemented with L-asparagine and phenol red. Talluri and co-workers (2013) isolated about 21 bacterial strains for L-asparaginase production from the soil samples using this method. Among them, BSH-3 isolate showed the maximum activity about 85.7 IU/mg by 24 h culture at 37°C [34]. Basha et al., (2009) isolated 10 strains which produced L-asparaginase with yields ranging from 24.61 to 49.23 U/ml [35]. Pradhan et al., (2013) identified *Bacillus subtilis* strain hswx88 with extracellular L-asparaginase production, yielding 23.8 IU/ml [36]. L-asparaginase activity of *Bacillus* sp. BCCS 034 was 1.64 IU/ml, as reported by Kumar and co-workers [37, 38]. Dhanam and Kannan (2014) isolated and characterized *actinomycetes* (*streptomyces*) from Periyar Lake, Kumily. These strains are the latest sources for high yield of producing L-asparaginase enzyme with high substrate specificity [39]. Reducing L-asparaginase activity in the presence of specific metal ions and the metal chelator, EDTA may be indicating that it was a metalloprotein. Thiol reactivity was also detected with the *E. carotovora* L-asparaginase. The inhibition of L-asparaginase activity in the presence of Hg^2+ ^revealing the presence of sulfhydryl group(s) [40]. It is mentioned that, hot springs are vigorous environments occupied by an enormous diversity of microbial populations. These environments encompass novel bacterial strains with novel properties of biotechnological attention. *P. pseudoalcaligenes* strain JHS-71 is a strain which produced L-asparaginase more than the other strains that has reported up to now. Suitable activity in the presence of some metal ions makes this enzyme as a potential candidate for biotechnological applications. 

## Notes

**Acknowledgements: **The author wishes to express his most gratitude to the Research Council of the Shahid Bahonar University of Kerman for financial support during the course of this project.
